# Risk factor analysis of fragility fractures in rheumatoid arthritis: A 3-year longitudinal, real-world, observational, cohort study

**DOI:** 10.1371/journal.pone.0255542

**Published:** 2021-08-04

**Authors:** Po-Heng Lin, Shan-Fu Yu, Jia-Feng Chen, Ying-Chou Chen, Han-Ming Lai, Wen-Chan Chiu, Chung-Yuan Hsu, Yu-Wei Wang, Hsiao-Ru He, You-Yin Chen, Chu-Yin Cheng, Tien-Tsai Cheng

**Affiliations:** 1 Department of Surgery, Kaohsiung Chang Gung Memorial Hospital, Kaohsiung, Taiwan; 2 Division of Rheumatology, Allergy, and Immunology, Department of Internal Medicine, Kaohsiung Chang Gung Memorial Hospital, Kaohsiung, Taiwan; 3 Department of Internal Medicine, Chang Gung University College of Medicine, Kaohsiung, Taiwan; 4 Department of Biomedical Engineering, National Yang Ming University, Taipei, Taiwan, Republic of China; 5 The Ph.D. Program for Neural Regenerative Medicine, College of Medical Science and Technology, Taipei Medical University, Taipei, Taiwan, Republic of China; 6 Department of Emergency Medicine, Kaohsiung Chang Gung Memorial Hospital, Kaohsiung, Taiwan; Medical College of Wisconsin, UNITED STATES

## Abstract

**Objectives:**

To explore the risk factors for fragility fractures in rheumatoid arthritis (RA) patients using a 3-year longitudinal, observational cohort study.

**Methods:**

This RA registry study included consecutive RA patients in the outpatient clinic of Chang Gung Memorial Hospital since September 1, 2014. The demographics, clinical characteristics, lifestyle, evidence of previous fracture, risk factors according to the Fracture Risk Assessment Tool (FRAX^®^), and the FRAX score of each participant were recorded. The participants were categorized into the new incident fracture (group A) and no incident fracture (group B) groups based on evidence or absence of new incident fractures and propensity score matching (age and gender, 1:2).

**Results:**

Overall, 477 participants completed the 3-year observation period. After matching, 103 and 206 participants were allocated to groups A and B, respectively. The non-adjusted model revealed, presented as hazard ratio (HR) (95% confidence interval [CI]), that the presence of co-morbidity (1.80 [1.17–2.78], p = 0.008), Health Assessment Questionnaire Disability Index (1.35 [1.07–1.69], p = 0.010), lower baseline hip bone mineral density (0.11 [0.02–0.48], p = 0.004), longer disease duration (1.02 [1.00–1.04], p = 0.026), higher FRAX score of major fracture (1.03 [1.02–1.04], p<0.001) or hip fracture (1.03 [1.02–1.04], p<0.001), and previous fracture history (2.65 [1.79–3.94], p<0.001) were associated with new incident fracture. After adjustment, it was disclosed that a previous fracture is an independent risk factor for fragility fractures in RA patients (2.17 [1.20–3.90], p = 0.010).

**Conclusions:**

In addition to aging and disease-related factors, previous fracture history is the most important risk factor for fragility fractures in RA patients.

## Introduction

Rheumatoid arthritis (RA) is a chronic, autoimmune, and systemic inflammatory disease characterized by inflammation of the peripheral joints and both local and systemic bone loss. RA patients have a higher risk of generalized osteoporosis due to systemic inflammation and medications used to treat RA, especially glucocorticoids.

In addition to osteoporosis, the risk of fragility fracture in RA patients is two- to three-fold of the general population [1]. Fragility fracture is not only associated with functional impairment and disability but is also associated with an immediate- and long-term risk of mortality [2]. Hence, RA is one of the major elements of the Fracture Risk Assessment (FRAX) tool that provides a 10-year fracture probability of fracture [3]. In the past decades, several medications, including conventional synthetic disease-modifying antirheumatic drugs (csDMARDs), biological disease-modifying antirheumatic drugs (bDMARDs), and targeted synthetic disease-modifying antirheumatic drugs (tsDMARDs), are available. These regimens not only demonstrated obvious effects on the control of disease activity and improvement of functional disability, but also revealed the efficacy of bone loss protection [4]. However, the incidence of non-vertebral fracture did not change in Japanese patients with RA [5] in the era of biologic application. This suggests that not only anti-osteoporosis medications (AOM) but also fragility fracture prevention should be addressed regardless of disease control in patients with RA.

A prospective, observational study investigating the predictors of refracture among RA patients managed within a secondary fracture prevention program revealed that poor compliance, multiple co-morbidities, glucocorticoid therapy, low hip bone mineral density (BMD), and low body weight were all significantly associated with refracture in patients who commenced long-term antiresorptive therapy [6]. However, the risk factors for fragility fracture in patients with real-world established RA, with or without fracture, remain unclear.

In the current investigation, we aimed to explore the risk factors for fragility fractures with a real-world, prospective, longitudinal, observational study on a cohort of patients with RA.

## Methods

### Study population and design

**Fig 1:**
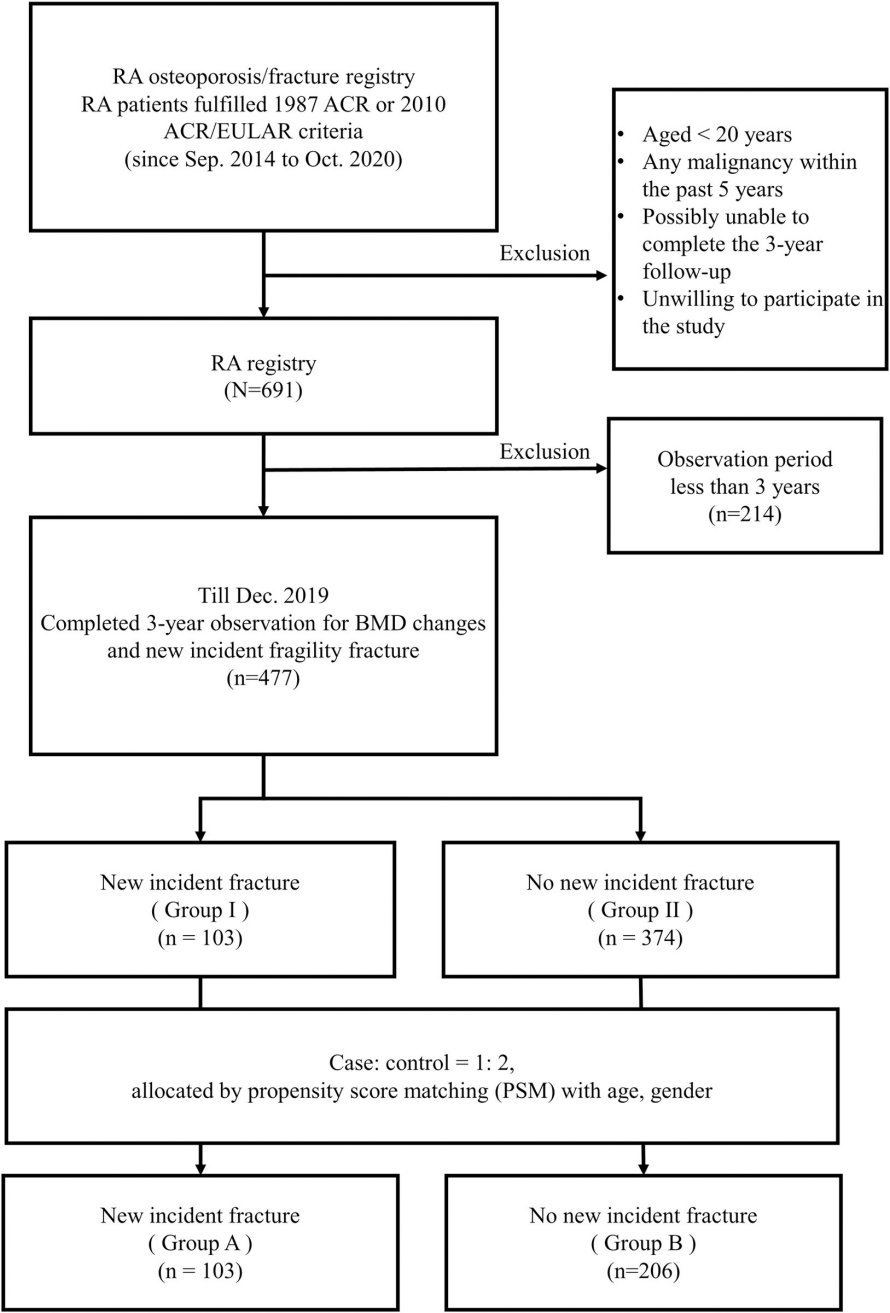
Disposition of the participants and grouping of participants.

The current investigation is an interim analysis of RA-related osteoporosis/fractures in an RA registry conducted at Chang Gung Memorial Hospital, Kaohsiung (CGMHK), Taiwan. Part of the study result has been presented and the inclusion criteria for participants have been previously published [7]. In brief, consecutive patients with RA who fulfilled the 1987 American College of Rheumatology (ACR) revised criteria [8] or the 2010 ACR/European League Against Rheumatism classification criteria [9] and who had visited the rheumatology clinic at CGMHK since September 1, 2014, were enrolled (Fig 1). The exclusion criteria were as follows: aged < 20 years, malignancy in the previous 5 years before enrollment, or unwilling to participate in the study. Informed consent was obtained from all subjects before study entry.

At enrollment, demographic data, including age, sex, body height, body weight, body mass index, positivity for anti-cyclic citrullinated peptide antibody (anti-CCP), rheumatoid factor, disease duration, co-morbidity and risk factors for fragility fractures in the FRAX^®^ tool were recorded. Disease duration was defined as the time elapsed between the onset of the first disease-related symptoms and enrollment. Co-morbidities of the participants were recorded based on Charlson Comorbidity Index with some minor modification, such as excluding malignancy variables. The variable “co-morbidity” indicates any presence of the collected co-morbidities. Evidence of previous fragility fractures was documented by reviewing patients electronic medical record through history and radiograph. We also recorded patients’ lifestyle, including vegan diet [10–12], smoking status, and daily coffee/tea consumption [13, 14], which are potentially related to disease activity and osteoporosis/fracture.

Besides, these assessments below were documented at enrollment and every 3–6 months. Baseline laboratory data, including biochemistry, hemogram, 25-hydroxyvitamin D3, and intact parathyroid hormone levels, as well as information on current medication, including csDMARD, bDMARD, tsDMARD, and the duration and dosage of glucocorticoids at enrollment of each participant were documented. Disease activity was evaluated according to the C-reactive protein level, erythrocyte sedimentation rate (ESR), and Disease Activity Score of 28 joints based on ESR (DAS28-ESR) at enrollment and during the observation period.

The primary outcome of the current study was to explore the risk factors for new incident fractures, including new clinical or morphometric fractures, of RA patients during the 3-year observation period. A new incident fracture was defined as any new symptomatic fragility fracture, including forearm, hip, pelvis, and humerus fracture or morphometric fracture. Morphometric fracture was defined as morphometry on spinal radiographs of lateral projection, following the visual semiquantitative diagnosis of an independent radiologist, according to Genant’s semiquantitative assessment of vertebral fractures [15]. An independent radiologist assessed the evidence of morphometric vertebral compression fracture at enrollment and subsequent, as needed basis, follow-up spinal radiographs during the 3-year observation period and at the end of the study. Therefore, in our study, 3 scenarios will be documented as new fracture incidents: Patient self-reported fracture confirmed by roentgenography or electronic health record during follow up; patient complained fracture related symptoms during follow up and fracture was confirmed by roentgenography; fracture diagnosed by routine roentgenography check-up at end of 3 year follow up.

All participants provided written informed consent. This study was approved by the local Institutional Review Board of Chang Gung Memorial Hospital (104-3530B, 201901054B0) and was performed according to the principles of the Declaration of Helsinki.

### Statistics

Student’s t-test and the Mann–Whitney U test were performed on the variables with a gaussian distribution. The variables are presented as means ± standard deviations. Nonparametric analysis was performed on variables with a skewed distribution, which are presented as medians (interquartile ranges). Categorical data were evaluated using the chi-square test or Fisher’s exact test and are presented as frequencies and percentages. P values < 0.05 were considered significant. To delineate the risk factors for fragility fracture besides age and gender, we performed propensity score matching (PSM) using age and gender as matching variables. The risk of fracture between the fracture group and the non-fracture group was estimated using the Kaplan–Meier method with the log-rank test. The start time of the model is set as the registration time and the stop time is assigned at the timing of new fracture incidents in three different scenarios mentioned in the previous paragraph. All covariates significant in the univariate analyses at the P = 0.10 level were included in the multivariate model. Hazard ratios and 95% confidence intervals (CIs) were calculated. Statistical analyses were performed using the Statistical Package for the Social Sciences (SPSS, version 22.0) and PSM with R programming (R, version 3.6.3).

## Results

### Demographics and clinical characteristics of participants before propensity score matching

A total of 477 participants completed the 3-year follow-up and were enrolled. The participants’ demographic and clinical characteristics are shown in Table 1 [16]. Incident fractures were found in 103 (21.6%) participants during the 3-year observation period, and participants were allocated to the new incident fracture group (group I). In this group, 92 patients (89.3%) were female, aged 63.0 (11.0) years, and disease duration was 14.0 (10.0) years. Compared with the no new incident fracture group (group II), participants in group I had significantly lower levels of BMD (g/cm^2^) (0.594 [0.151] vs. 0.632 [0.146], p = 0.002; 0.747±0.147 vs. 0.797 [0.177], p = 0.002; 0.811 [0.233] vs. 0.867 [0.183], p = 0.021) at the femoral neck (FN), hip (total), and L1–L4, respectively. In addition, participants in group I revealed significantly higher Health Assessment Questionnaire Disability Index (HAQ-DI) scores (0.5 [1.688] vs. 0.250 [0.875], p < 0.001), higher rate of co-morbidity (75 [72.8%] vs. 208 (55.6%), p = 0.002), lower rate of tea consumption (12 [11.7%] vs. 76 [20.3%], p = 0.045), and higher total bilirubin levels (0.7 [0.3] vs. 0.6 [0.3], p = 0.019). Besides, in group I, 62 participants (60.2%) were treated with AOM and in group II, there were 97 participants (25.9%). (Tables 1 and S1).

**Table 1:** Demographics and clinical characteristics of participants before PSM.

Demographics	Total (n = 477)	Group I (n = 103)	Group II (n = 374)	p
**Age (years old)**	59.0 (14.0)	62.7±8.9	57.0 (14.0)	<0.001*
**Female, n (%)**	406 (85.1)	92(89.3)	314 (84.0)	
**Body height (cm)**	156.8±7.4	155.7±7.2	157.1±7.4	
**Body weight (kg)**	57.0 (13.8)	56.3 (14.7)	57.1 (13.5)	
**BMI (kg/m**^**2**^**)**	23.2 (5.1)	23.6 (4.6)	23.2 (5.1)	
**Vegan diet +, n (%)**	26 (5.5)	9 (8.7)	17 (4.5)	
**Tea +, n (%)** ^**a**^	88 (18.4)	12 (11.7)	76(20.3)	0.045*
**Coffee +, n (%)** ^**a**^	76 (15.9)	11(10.7)	65 (17.4)	
**History of fall +, n (%)** ^**b**^	80 (17.2)	17(17.3)	63(17.1)	
**Co-morbidity +, n (%)**	283 (59.3)	75 (72.8)	208 (55.6)	0.002*
**b/ts DMARDs +, n (%)** ^**c**^	83 (17.4)	14 (13.6)	69 (18.5)	
**Disease duration (years)** ^**d**^	12.0 (13.0)	14.0 (10.0)	12.0 (13.0)	<0.001*
**RA related factors**				
**CRP tertials, n (%)** ^**e**^				
**I**	160 (33.5)	37 (35.9)	123 (32.9)	
**II**	159 (33.3)	31(30.1)	128 (34.2)	
**III**	158 (33.1)	35(34.0)	123 (32.9)	
**Anti-CCP +, n (%)**	320 (67.9)	70 (68.6)	250 (67.8)	
**RF +, n (%)**	300 (65.6)	70 (70.0)	230 (64.4)	
**ESR (mm/hr)**	17.0 (21.0)	19.0 (23.0)	16.0 (20.3)	
**DAS-28 (ESR)**	3.0 (1.2)	3.3±1.0	3.0 (1.2)	
**HAQ-DI**	0.3 (1.0)	0.5 (1.7)	0.3 (0.9)	<0.001*
**BMD (g/cm**^**2**^**)**				
**Femoral neck**	0.623 (0.141)	0.594 (0.151)	0.632 (0.146)	0.002*
**Hip (total)**	0.786±0.140	0.747±0.147	0.797 (0.177)	0.002*
**L1~L4**	0.858 (0.200)	0.811 (0.233)	0.867 (0.183)	0.021*
**AOM** ^**f**^**, n (%)**	159 (33.3)	62 (60.2)	97 (25.9)	<0.001*
**FRAX** ^**g**^**, n (%)**				
**Glucocorticoid +**	416 (87.4)	95 (92.2)	321 (86.1)	
**Current Smoking +**	31 (65)	8 (7.8)	23 (6.1)	
**Alcohol 3 or more units/day +**	7 (1.5)	3 (2.9)	4 (1.1)	
**Previous fracture +**	151 (31.7)	62 (60.2)	89 (23.8)	<0.001*
**Parent fractured hip +**	37 (7.8)	9 (8.9)	28 (7.5)	
**Secondary osteoporosis +**	2 1(4.4)	4 (3.9)	17 (4.5)	
**FRAX score** ^**h**^				
**Major**	14.0 (18.7)	28.0 (24.5)	12.0 (13.7)	<0.001*
**Hip**	4.5 (9.3)	11.0 (15.4)	3.6 (6.9)	<0.001*

BMI, body mass index; anti-CCP, anti-cyclic citrullinated peptide antibody; RF, rheumatoid factor; CRP, C-reactive protein; ESR, erythrocyte sedimentation rate; DAS28-ESR, Disease Activity Score of 28 joints using ESR; HAQ-DI, Health Assessment Questionnaire Disease Index; BMD, bone mineral density; L1–L4, first to fourth segment of the lumbar spine.

^a^ Tea/coffee consumption: daily consumption of more than or equal to 1 cup/day.

^b^ History of fall: had history of fall within 1 year prior to registration.

^c^ b/tsDMARDs: in the current study, b/tsDMARDs included anti-TNFa (etanercept, adalimumab, golimumab, certolizumab), anti-IL6 receptor (tocilizumab), CTLA4 analog (abatacept), anti-CD 20 (rituximab), and JAK inhibitor (tofacitinib).

^d^ From diagnosis of disease to registration.

^e^ Subgrouped by tertials: I (<0.02–1.1), II (1.2–4.80), III (>4.80).

^f^ AOM: anti-osteoporosis medications, including Bisphosphonate (Alendronate, Ibandronic acid, Zoledronic acid), RANK ligand (RANKL) inhibitor (Denosumab), Estrogen and Selective estrogen receptor modulators (Raloxifene).

^g^ FRAX: risk factors for fragility fracture as defined in the FRAX tool.

^h^ FRAX score: 10-year probability of fracture.

### Risk factor analysis of new incident fracture using the Cox proportional hazard model before PSM

The univariate analysis (Table 2), presented as HR (95% CI), revealed that aging (1.06 [1.04–1.08], p < 0.001), presence of co-morbidity (1.99 [1.29–3.08], p = 0.002), higher HAQ-DI score (1.72 [1.37–2.15], p < 0.001), lower BMD at the FN (0.06 [0.01–0.39], p = 0.003) and hip (total) (0.08 [0.02–0.35], p = 0.001), longer disease duration (1.03 [1.01–1.05], p = 0.003), and FRAX score, either major (1.05 [1.04–1.06], p < 0.001) or hip fracture (1.04 [1.03–1.06], p < 0.001), were associated with new incident fractures. After adjustment, the presence of co-morbidity (1.70 [1.02–2.83], p = 0.041) and previous fracture (1.95 [1.06–3.56], p = 0.031) were independently associated with new incident fracture.

**Table 2:** Risk factor analysis of new incident fracture before PSM.

Variables ^a^	Uni-variable			Multi-variable		
Demographics	beta	HR (95% CI)	P	beta	HR (95% CI)	P
**Age**	0.055	1.06 (1.04–1.08)	<0.001	0.010	1.010 (0.979–1.041)	0.549
**Female**	-0.421	0.66 (0.35–1.23)	0.187			
**Body height**	-0.022	0.98 (0.95–1.00)	0.099	-0.001	0.999 (0.964–1.035)	0.958
**Body weight**	-0.008	0.99 (097–1.01)	0.347			
**BMI**	-0.009	0.99 (0.94–1.04)	0.725			
**Vegan diet**	0.657	1.93 (0.97–3.82)	0.060	0.698	2.010 (0.901–4.484)	0.088
**Tea** ^**b**^	-0.596	0.55 (0.30–1.01)	0.052	-0.122	0.885 (0.460–1.703)	0.715
**Coffee** ^**b**^	-0.504	0.60 (0.32–1.13)	0.114			
**History of fall** ^**c**^	0.022	1.02 (0.61–1.73)	0.934			
**Co-morbidity**	0.689	1.99 (1.29–3.08)	0.002	0.530	1.699 (1.021–2.828)	0.041
**b/ts DMARDs** ^**d**^	-0.314	0.73 (0.42–1.28)	0.275			
**Disease duration** ^**e**^	0.029	1.03 (1.01–1.05)	0.003	0.009	1.009 (0.984–1.034)	0.470
**RA related factors**						
**CRP tertials** ^**f**^						
**I**		ref				
**II**	-0.194	0.82 (0.51–1.33)	0.425			
**III**	-0.050	0.95 (0.60–1.51)	0.833			
**Anti-CCP**	0.040	1.04 (0.69–1.57)	0.853			
**RF**	0.239	1.27 (0.83–1.95)	0.273			
**ESR**	0.004	1.00 (1.00–1.01)	0.353			
**DAS-28 (ESR)**	0.209	1.23 (1.01–1.50)	0.036	0.002	1.002 (0.779–1.288)	0.988
**HAQ-DI**	0.539	1.72 (1.37–2.15)	<0.001	0.206	1.228 (0.898–1.680)	0.199
**BMD**						
**Femoral neck**	-2.753	0.06 (0.01–0.39)	0.003	-2.678	0.069 (0.001–3.632)	0.186
**Hip (total)**	-2.523	0.08 (0.02–0.35)	0.001	-0.337	0.714 (0.028–18.162)	0.838
**L1~L4**	-1.043	0.35 (0.11–1.18)	0.090	0.853	2.347 (0.442–12.469)	0.317
**FRAX** ^**g**^						
**Glucocorticoid**	0.601	1.82 (0.89–3.75)	0.103			
**Current Smoking**	0.190	1.21 (0.59–2.49)	0.606			
**Alcohol 3 or more units/day**	0.794	2.21 (0.70–6.98)	0.175			
**Previous fracture**	1.365	3.92 (2.64–5.82)	<0.001	0.665	1.95 (1.06–3.56)	0.031
**Parent fractured hip**	0.140	1.15 (0.58–2.28)	0.689			
**Secondary osteoporosis**	-0.130	0.88 (0.32–2.39)	0.799			
**FRAX score** ^**h**^						
**Major**	0.045	1.05 (1.03–1.06)	<0.001	0.040	1.040 (0.990–1.093)	0.118
**Hip**	0.043	1.04 (1.03–1.06)	<0.001	-0.019	0.981(0.933–1.032)	0.460

BMI, body mass index; anti-CCP, anti-cyclic citrullinated peptide antibody; RF, rheumatoid factor; CRP, C-reactive protein; ESR, erythrocyte sedimentation rate; DAS28-ESR, Disease Activity Score of 28 joints using ESR; HAQ-DI, Health Assessment Questionnaire Disease Index; BMD, bone mineral density; L1–L4, first to fourth segment of the lumbar spine.

^a^ Defined as in Table 1, per unit increase or positive (+) vs. negative (-).

^b^ Tea/coffee consumption: daily consumption of more than or equal to 1 cup/day.

^c^ History of fall: had history of fall within 1 year prior to registration.

^d^ b/ts DMARDs: including anti-TNFa (etanercept, adalimumab, golimumab, certolizumab), anti-IL6 receptor (tocilizumab), CTLA4 analog (abatacept), anti-CD 20 (rituximab), and JAK inhibitor (tofacitinib).

^e^ From diagnosis of RA to date of registration.

^f^ Subgrouped by tertials: I (<0.02–1.1), II (1.2–4.80), III (>4.80).

^g^ FRAX: risk factors for fragility fracture as defined in the FRAX tool.

^h^ FRAX score: 10-year probability of fracture.

### Demographics and clinical characteristics of participants after PSM

After PSM for age and gender (1:2), 103 and 206 participants were allocated to the new incident fracture group (group A) and no incident fracture group (group B), respectively (Table 3) [17]. Compared to group B, the participants in group A had a significantly higher rate of co-morbidity (n, %) (75 [72.8] vs. 119 [57.8], p = 0.012), higher FRAX score (%) of major fracture (28.0 [24.5] vs. 18.0 [17.8], p < 0.001) or hip fracture (11.0 [15.4] vs. 5.7 [9.0], p < 0.001), and higher proportion (%) of previous fracture (62 [60.2] vs. 58 [28.2], p < 0.001). As for AOM, in group A, there remain 62 participants (60.2%) receiving treatment while in group B, there were 71 participants (34.5%). (Table 3).

**Table 3:** Demographics and clinical characteristics of participants after PSM.

Demographics	Total (n = 309)	Group A (n = 103)	Group B (n = 206)	p
**Age (years old)**	63.0 (10.0)	62.7±8.9	63.0 (10.0)	
**Female, n (%)**	273(88.3)	92(89.3)	181(87.9)	
**Body height (cm)**	156.6±7.5	155.7±7.2	157.0±7.6	
**Body weight (kg)**	56.3 (13.7)	56.3 (14.7)	56.4 (13.5)	
**BMI (kg/m**^**2**^**)**	23.2 (5.2)	23.6 (4.6)	23.1 (5.5)	
**Vegan diet +, n (%)**	23 (7.4)	9 (8.7)	14 (6.8%)	
**Tea +, n (%)** ^**a**^	47 (15.2)	12 (11.7)	35 (17.0%)	
**Coffee +, n (%)** ^**a**^	44 (14.2)	11 (10.7)	33 (16.0%)	
**History of fall +, n (%)** ^**b**^	54 (17.9)	17 (17.3)	37 (18.2%)	
**Co-morbidity +, n (%)**	191 (61.8)	75 (72.8)	116 (56.3%)	0.005*
**b/ts DMARDs +, n (%)** ^**c**^	52 (16.9)	14 (13.6)	38 (18.5)	
**Disease duration (year)** ^**d**^	13.0 (12.3)	14.0 (10.0)	12.0 (13.0)	0.001*
**RA related factors**				
**CRP tertials, n (%)** ^**e**^				
**I**	110 (35.6)	37 (35.9)	73 (35.4)	
**II**	100 (32.4)	31 (30.1)	69 (33.5)	
**III**	99 (32.0)	35 (34.0)	64 (31.1)	
**Anti-CCP +, n (%)**	200 (65.4)	70 (68.6)	130 (63.7)	
**RF +, n (%)**	194 (64.9)	70 (70.0)	124 (62.3)	
**ESR (mm/hr)**	17.0 (19.5)	19.0 (23.0)	16.0 (19.3)	
**DAS-28 (ESR)**	3.0 (1.2)	3.1 (1.4)	3.0 (1.1)	
**HAQ-DI**	0.4 (1.1)	0.5 (1.7)	0.4 (1.0)	0.037*
**BMD (g/cm**^**2**^**)**				
**Femoral neck**	0.618 (0.140)	0.594 (0.150)	0.630 (0.140)	0.002*
**Hip (total)**	0.776 (0.170)	0.747±0.147	0.795 (0.170)	0.005*
**L1~L4**	0.854 (0.200)	0.811 (0.230)	0.867 (0.180)	0.019*
**AOM** ^**f**^	133 (43.0)	62 (60.2)	71 (34.5)	<0.001*
**FRAX** ^**g**^**, n (%)**				
**Glucocorticoid +**	272 (88.0)	95 (92.2)	177 (85.9)	
**Current Smoking +**	19 (6.1)	8 (7.8)	11 (5.3)	
**Alcohol 3 or more units/day +**	5 (1.6)	3 (2.9)	2 (1.0)	
**Previous fracture +**	122 (39.5)	62 (60.2)	60 (29.1)	<0.001*
**Parent fractured hip +**	26 (8.5)	9 (8.9)	17 (8.3)	
**Secondary osteoporosis +**	16(5.2)	4 (3.9)	12 (5.8)	
**FRAX score** ^**h**^				
**Major**	21.0 (19.0)	28.0 (24.50)	18.0 (17.75)	<0.001*
**Hip**	7.1 (10.9)	11.0 (15.40)	5.7 (9.0)	<0.001*

BMI, body mass index; anti-CCP, anti-cyclic citrullinated peptide antibody; RF, rheumatoid factor; CRP, C-reactive protein; ESR, erythrocyte sedimentation rate; DAS28-ESR, Disease Activity Score of 28 joints using ESR; HAQ-DI, Health Assessment Questionnaire Disease Index; BMD, bone mineral density; L1–L4, first to fourth segment of the lumbar spine.

^a^ Tea/coffee consumption: daily consumption of more than or equal to 1 cup/day.

^b^ History of fall: had history of fall within 1 year prior to registration.

^c^ b/ts DMARDs: in the current study, b/tsDMARDs include anti-TNFa (etanercept, adalimumab, golimumab, certolizumab), anti-IL6 receptor (tocilizumab), CTLA4 analog (abatacept), anti-CD 20 (rituximab), and JAK inhibitor (tofacitinib).

^d^ From diagnosis of disease to registration.

^e^ Subgrouped by tertials: I (<0.02–1.1), II (1.2–4.80), III (>4.80).

^f^ AOM: anti-osteoporosis medications, including Bisphosphonate (Alendronate, Ibandronic acid, Zoledronic acid), RANK ligand (RANKL) inhibitor (Denosumab), Estrogen and Selective estrogen receptor modulators (Raloxifene).

^g^ FRAX: risk factors for fragility fracture as defined in the FRAX tool.

^h^ FRAX score: 10-year probability of fracture.

### Risk factor analysis of new incident fracture using the Cox proportional hazard model after PSM

**Fig 2:**
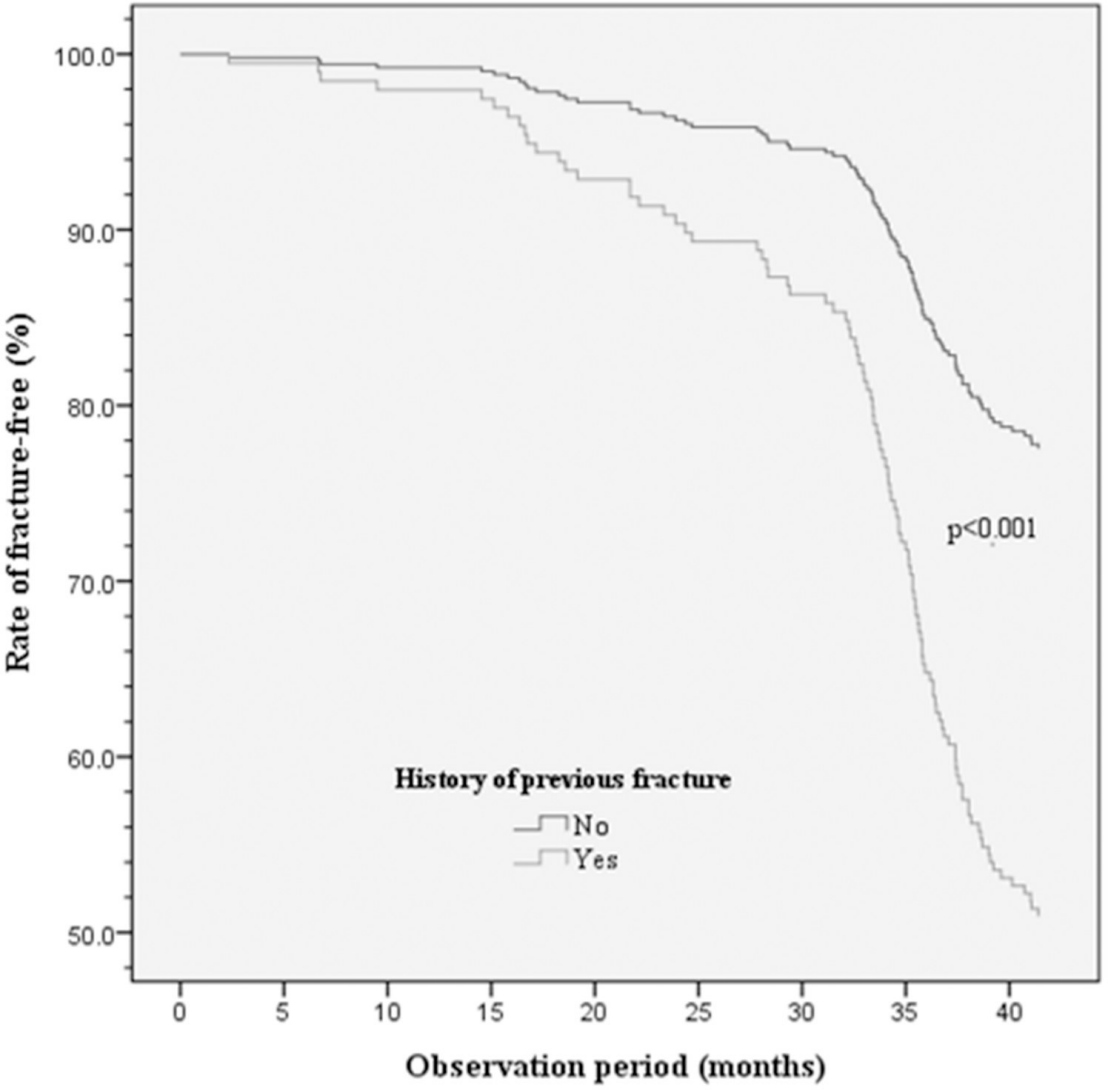
Impact of the history of a previous fracture on new fractures.

The univariate analysis, presented as HR (95% CI) (Table 4), revealed that the presence of co-morbidity (1.80 [1.17–2.78], p = 0.008), higher HAQ-DI score (1.35 [1.07–1.69], p = 0.010), lower baseline hip BMD (0.11 [0.02–0.48], p = 0.004), longer disease duration (1.02 [1.00–1.04], p = 0.026), higher FRAX score of major fracture (1.03 [1.02–1.04], p < 0.001) or hip fracture (1.03 [1.02–1.04], p < 0.001), and a history of previous fracture (2.65 [1.79–3.94], p < 0.001) were associated with new incident fracture after 3 years. After adjustment, it was disclosed that previous fracture is the single risk factor of fragility fracture in our cohort (2.17 [1.20–3.90], p = 0.010) (Table 4 and Fig 2).

**Table 4:** Risk factor analysis of new incident fracture after PSM.

Variables ^a^	Uni-variable			Multi-variable		
Demographics	beta	HR (95%CI)	P	beta	HR (95%CI)	P
**Age**	0.007	1.01 (0.99–1.03)	0.529			
**Female**	-0.117	0.89 (0.48–1.66)	0.890			
**Body height**	-0.017	0.98 (0.96–1.01)	0.194			
**Body weight**	-0.004	1.00 (0.98–1.01)	0.635			
**BMI**	<0.001	1.00 (0.96–1.04)	0.994			
**Vegan diet**	0.360	1.43 (0.72–2.84)	0.303			
**Tea** ^**b**^	-0.180	0.84 (0.46–1.52)	0.557			
**Coffee** ^**b**^	-0.349	0.71 (0.38–1.32)	0.274			
**History of fall** ^**c**^	-0.036	0.97 (0.57–1.63)	0.892			
**Co-morbidity**	0.589	1.80 (1.17–2.78)	0.008	0.370	1.448 (0.876–2.394)	0.148
**b/ts DMARDs** ^**d**^	-0.226	0.80 (0.45–1.40)	0.433			
**Disease duration (years)** ^**e**^	0.022	1.02 (1.00–1.04)	0.026	0.014	1.014 (0.990–1.039)	0.246
**RA related factors**						
**CRP tertials** ^**f**^						
**I**		ref				
**II**	-0.172	0.84 (0.52–1.36)	0.480			
**III**	0.063	1.07 (0.67–1.69)	0.791			
**Anti-CCP**	0.080	1.08 (0.71–1.65)	0.708			
**RF**	0.247	1.28 (0.83–1.96)	0.259			
**ESR**	0.005	1.01 (1.00–1.01)	0.252			
**DAS-28 (ESR)**	0.133	1.14 (0.94–1.39)	0.189			
**HAQ-DI**	0.298	1.35 (1.07–1.69)	0.010	0.112	1.119 (0.855–1.464)	0.413
**BMD (g/cm**^**2**^**)**						
**Femoral neck**	-2.346	0.10 (0.02–0.59)	0.096	-2.245	0.106 (0.002–5.007)	0.254
**Hip (total)**	-2.249	0.11 (0.02–0.48)	0.004	-0.436	0.647 (0.025–16.852)	0.793
**L1~L4**	-0.827	0.44 (0.13–1.44)	0.173	0.266	1.305 (0.259–6.571)	0.747
**FRAX** ^**g**^						
**Glucocorticoid**	0.432	1.54 (0.75–3.17)	0.241			
**Current Smoking**	0.409	1.51 (0.73–3.10)	0.367			
**Alcohol 3 or more units/day**	1.057	2.88 (0.91–9.08)	0.072	0.864	2.372 (0.631–8.915)	0.201
**Previous fracture**	0.976	2.65 (1.79–3.94)	<0.001	0.773	2.167 (1.203–3.902)	0.010
**Parent fractured hip**	-0.026	0.97 (0.49–1.93)	0.940			
**Secondary osteoporosis**	-0.334	0.72 (0.26–1.95)	0.512			
**FRAX score** ^**h**^						
**Major**	0.029	1.03 (1.02–1.04)	<0.001	-0.010	0.991 (0.944–1.040)	0.699
**Hip**	0.029	1.03 (1.02–1.04)	<0.001	0.023	1.023 (0.974–1.075)	0.365

BMI, body mass index; anti-CCP, anti-cyclic citrullinated peptide antibody; RF, rheumatoid factor; CRP, C-reactive protein; ESR, erythrocyte sedimentation rate; DAS28-ESR, Disease Activity Score of 28 joints using ESR; HAQ-DI, Health Assessment Questionnaire Disease Index; BMD, bone mineral density; L1–L4, first to fourth segment of the lumbar spine.

^a^ Defined as in Table 3, per one unit increase or positive (+) vs. negative (-).

^b^ Tea/coffee consumption: daily consumption of more than or equal to 1 cup/day.

^c^ History of fall: having history of fall within 1 year prior to registration.

^d^ b/ts DMARDs: including anti-TNFa (etanercept, adalimumab, golimumab, certolizumab), anti-IL6 receptor (tocilizumab), CTLA4 analog (abatacept), anti-CD 20 (rituximab), and JAK inhibitor (tofacitinib).

^e^ From diagnosis of RA to date of registration.

^f^ Subgrouped by tertials: I (<0.02–1.1), II (1.2–4.80), III (>4.80).

^g^ FRAX: risk factors for fragility fracture as defined in the FRAX tool.

^h^ FRAX score: 10-year probability of fracture.

## Discussion

The current investigation, before PSM, revealed that aging, presence of co-morbidity, higher baseline disease activity (DAS28-ESR), higher HAQ-DI levels, longer disease duration, lower FN and hip (total) BMD levels, and history of previous fracture were associated with new incident fracture in our cohort. After adjustment, co-morbidity and previous fracture are two independent risk factors for incident fracture after 3 years of observation. Before PSM analysis, we found that the age of the fracture group (Group I) was significantly higher (62.7±8.9 and 57.0 (14.0), p <0.001) than non-fracture group (Group II). In addition, the findings in univariate survival analysis revealed several factors associated with aging, including age itself, disease duration, bone mineral density (BMD), as well as co-morbidity. Age and aging related confounding factors are well-known risk factors of fragility fracture in RA patients. In order to investigate the other possible risk factors other than age, we did the propensity score matching (PSM) to minimize the influence of age and aging related factors. After PSM and multi-variate analysis, we found that the history of previous fracture was the single independent risk factor of incident fracture in RA patients.

The FRAX^®^ algorithms provide the 10-year probability of a fracture. The output is the 10-year probability of hip fracture and the 10-year probability of a major osteoporotic fracture. The algorithms include the factor of RA and allow the health-care provider to estimate the 10-year fracture risk of RA patients. Several risk factors, including glucocorticoid use [18, 19], medications [20, 21], anti-CCP positivity [22], disease duration [23–25], higher HAQ-DI levels [26], lower hip BMD, and co-morbidity [6], of fragility fractures in patients with RA have been proposed in previous investigations. However, most of the aforementioned risk factors, except glucocorticoid use [20, 25], of fragility fractures were not included specific for RA patients in the estimation of fragility fracture in FRAX^®^. In terms of precision medicine, therefore, it is mandatory to prospectively explore the risk factors, other than the elements in the FRAX^®^ tool, of fragility fractures for RA.

Although several risk factors have been identified in previous studies, previous investigations were subjected to either cross-sectional study [24, 25], retrospective observation [1], limited variables included [25, 26], small sample size [20] or self-reported outcome [27]. In our investigation, we included demographics, variables related to disease entity, lifestyle, medications used, fall history, and fracture risk factors in the FRAX^®^ tool that could be related to osteoporosis/fracture in our cohort to explore the possible risk factors for fracture in patients with RA. Since the advent of the FRAX^®^ tool, fracture prediction has never been validated in a prospective study specific for RA patients. In the current investigation, regardless of PSM, the FRAX score (major or hip fracture) was significantly higher in the new incident fracture group than in the no new incident fracture group. This suggests that the FRAX score could also predict RA-related fragility fractures even after a 3-year observation period. Consistent with the factors in the FRAX^®^ tool, the current investigation revealed that aging, lower BMD levels, and previous fractures are also risk factors for fragility fractures in RA. Other than the risk factors in the FRAX^®^ tool, we found that the disease entity of RA, for example, presence of co-morbidity, disease activity (DAS28-ESR), higher HAQ-DI levels and longer disease duration are associated with fracture risk. As aging is one of the major determinants in the evaluation of fracture risk, and aging-related confounding factors may also influence the risk factor analysis, we performed a PSM analysis to find additional factors that could be associated with RA-related fractures. After matching, the presence of co-morbidity, higher HAQ-DI levels, lower hip BMD, longer disease duration, previous fracture, and FRAX^®^ score (major or hip fracture) were the risk factors for new incident fracture for RA patients (Table 4). This finding is partially consistent with a longitudinal observation study by Ganda K et al. [6], which indicated that comorbidities and low hip BMD were the risk factors for refracture. However, this was the case in our series, as participants enrolled in the current investigation were established RA either with or without fracture. Compared with the study by Ganda K et al.[6], we identified that higher baseline HAQ-DI levels, longer disease duration, and previous fracture are additional risk factors in this longitudinal observational study.

In terms of risk factors related to RA disease, it seems that disease duration [23–25] and higher HAQ-DI levels [26] were the most consistent risk factors for RA-related fracture in previous publications and also in the current series. A positive anti-CCP was implicated as a risk factor for fracture [7, 22]; however, in the current study, we could not demonstrate that a positive anti-CCP is associated with incident fracture in real-world practice.

In terms of risk factors for fragility fracture in the FRAX^®^ tool, before PSM, only aging, BMD at FN and hip (total), and previous fracture, but not glucocorticoid use, were associated with incident fracture. Our finding indicating that glucocorticoid use is not associated with new incident fractures is not consistent with previous investigations [18, 19]. We speculate that two possible mechanisms may play a role in explaining this finding. First, glucocorticoid use may suppress inflammation and counterbalance their adverse effects on bone remodeling [28]. Second, b/tsDMARDs were used in 73 (17.4%) patients in our cohort that not only had a bone loss protection effect [4, 29] but also the HAQ-DI improvement effect. Both could abrogate the detrimental effect on BMD and bone quality, which may indirectly reduce the fracture rate in our cohort.

After PSM and adjustment, we found that previous fracture was the sole independent risk factor of fragility in our cohort (Table 4 and Fig 2). It complies the notion of “one fracture predicts another fracture” [30, 31]. Our findings suggest that RA patients with a history of fragility fracture need intensive care or pharmacological intervention to prevent future fragility fractures.

The strength of the present study is that it is a 3-year real-world, longitudinal cohort study on fracture risk in RA patients, instead of a cross-sectional investigation. As a real-world investigation, we documented as many reported clinical variables that may be associated with osteoporosis/fractures in patients with RA to avoid missing the possible risk factors that were not observed in previous investigations. In addition, we defined incident fracture as any new clinical or morphometric vertebral fracture, proved by roentgenography and an independent radiologist at baseline and thereafter. Vertebral fractures are prevalent and often are asymptomatic [32]. Vertebral fractures are associated with functional impairment, higher mortality, and future vertebral and non-vertebral fractures independent of other clinical risk factors and BMD [2, 33]. However, previous investigations may ignore the asymptomatic vertebral fracture in the survey of incident fractures [6, 23] that could underestimate the rate of incident fracture and mislead risk factor analysis. Finally, in the current investigation, we performed a PSM for participants and allocated the participants into groups A and B to exclude age- or gender-related risk factors that were not performed in most previous investigations for risk factor exploration.

The current study has some limitations. This study is an interim analysis of RA-related osteoporosis/fracture registry. We completed 3-year observation for only 477 participants until the end of 2019. In terms of statistics, as the variables included in this study were multiple, we need to enroll more participants and observe longer periods of time to more clearly elucidate the risk factors for RA-related fragility fractures. However, the sample size of the current investigation is relatively large (n = 477) in a real-world, prospective, longitudinal, observational study, compared with other longitudinal investigations [6]. In addition, baseline FRAX^®^ scores of participants, both major and hip fractures, were significantly higher in group A participants; however, the FRAX^®^ tool provides the 10-year probability of a fracture. Whether the FRAX^®^ tool could precisely determine the 10-year probability of a fracture specific for RA patients via this 3-year observation study requires further investigation.

In summary, in addition to aging and aging-related factors such as co-morbidity and BMD, after PSM, we found that BMD at the hip, higher HAQ-DI score, longer disease duration, and previous fracture are the major risk factors for fracture. Among the risk factors, previous fractures constitute the single most important risk factor. In this study, we suggest that if health-care providers evaluate the fracture risk for RA patients, the aforementioned risk factors should be considered in addition to the application of the FRAX^®^ tool. Furthermore, we also recommend that RA patients with previous fragility fracture history need aggressive intervention to prevent future fragility fractures and fracture-related morbidity/mortality.

## Supporting information

S1 Table Laboratory data before PSM and after PSM. (DOCX)

S2 Table The prevalence of co-morbidity in participants before PSM. (DOCX)

S3 Table The prevalence of co-morbidity in participants after PSM. (DOCX)

S4 Table Missing data. (DOCX)
