# Supplementary material for: Risk factor analysis of fragility fractures in rheumatoid arthritis: A 3-year longitudinal, real-world, observational, cohort study
Source: PLoS One. 2021 Aug 4;16(8):e0255542. doi: 10.1371/journal.pone.0255542 (PMC8336806; doi:10.1371/journal.pone.0255542)
Supplement: S1 Table — (DOCX) [file pone.0255542.s001.docx]

S1 Table. Laboratory data before PSM and after PSM

Before PSM (missing data excluded)

*, defined as in Fig. 1

| Variables | Total | Group I* | Group II* | p |
| --- | --- | --- | --- | --- |
| 25(OH) Vit D (ng/ml) | 21.60(9.70) | 23.5±7.37 | 21.0(9.05) | 0.075 |
| iPTH (pg/ml) | 40.60(26.80) | 42.15(25.30) | 39.30(26.60) | 0.154 |
| Calcium (mg/dL) | 9.30(0.50) | 9.19(0.50) | 9.30(0.60) | 0.533 |
| P (mg/dL) | 3.71±0.53 | 3.72±0.57 | 3.7(0.8) | 0.945 |
| BUN (mg/dL) | 14.0(5.0) | 15.0(6.0) | 14.0(5.0) | 0.175 |
| Creatinine (mg/dL) | 0.67(0.22) | 0.70(0.23) | 0.67(0.22) | 0.836 |
| Albumin(g/dL) | 4.35±0.28 | 4.32(0.36) | 4.36(0.35) | 0.407 |
| ALT (U/L) | 21.0(18.0) | 21.0(17.0) | 21.0(18.0) | 0.932 |
| AST (U/L) | 22.0(10.0) | 23.0(11.0) | 22.0(11.0) | 0.351 |
| ALK-P (U/L) | 68.0(27.0) | 73.0(25.5) | 67.0(26.0) | 0.025 |
| Bil-T (mg/dL) | 0.60(0.40) | 0.70(0.30) | 0.60(0.30) | 0.019 |
| WBC (1000/μL) | 6.80(2.50) | 6.80(2.3) | 6.80(2.60) | 0.333 |
| Hemoglobin (g/dL) | 13.0(1.70) | 13.1(1.6) | 13.0(1.7) | 0.705 |
| Platelet (1000/μL) | 240.0(86.0) | 235.2±67.2 | 242.0(90.5) | 0.067 |

25(OH)D, 25-hydroxyvitamin D; iPTH, intact parathyroid hormone; P, phosphate; BUN, blood urea nitrogen; AST, aspartate transaminase; ALT, alanine aminotransferase; ALK-P, alkaline phosphatase; Bil-T, total bilirubin

After PSM (missing data excluded)

*, defined as in Fig. 1

| Variables | Total | Group A* | Group B* | p |
| --- | --- | --- | --- | --- |
| 25(OH) Vit D (ng/ml) | 21.45(9.20) | 23.53±7.37 | 23.30(10.45) | 0.025 |
| iPTH (pg/ml) | 41.60(26.15) | 42.15(25.30) | 40.25(25.92) | 0.277 |
| Calcium (mg/dL) | 9.30(0.50) | 9.28±0.39 | 9.30(0.50) | 0.626 |
| P (mg/dL) | 3.71±6.31 | 3.72±0.57 | 3.71±0.51 | 0.894 |
| BUN (mg/dL) | 15.0(5.0) | 15.0(6.0) | 14.0(5.0) | 0.268 |
| Creatinine (mg/dL) | 0.68(0.21) | 0.70(0.23) | 0.67(0.20) | 0.873 |
| Albumin(g/dL) | 4.36(0.36) | 4.33±0.29 | 4.37±0.27 | 0.224 |
| ALT (U/L) | 21.0(19.0) | 21.0(17.0) | 21.0(20.0) | 0.995 |
| AST (U/L) | 22.0(11.0) | 23.0(11.0) | 22.0(12.0) | 0.414 |
| ALK-P (U/L) | 69.0(27.0) | 73.0(25.50) | 67.0(26.25) | 0.045 |
| Bil-T (mg/dL) | 0.60(0.30) | 0.70(0.30) | 0.60(0.30) | 0.014 |
| WBC (1000/μL) | 6.70(2.55) | 6.80(2.30) | 6.60(2.63) | 0.199 |
| Hemoglobin (g/dL) | 13.0(1.70) | 13.10(1.60) | 12.95(1.72) | 0.676 |
| Platelet (1000/μL) | 238.0(89.0) | 235.23±67.42 | 239.5(93.5) | 0.279 |

25(OH)D, 25-hydroxyvitamin D; iPTH, intact parathyroid hormone; P, phosphate; BUN, blood urea nitrogen; AST, aspartate transaminase; ALT, alanine aminotransferase; ALK-P, alkaline phosphatase; Bil-T, total bilirubin
