# Supplementary material for: Risk factor analysis of fragility fractures in rheumatoid arthritis: A 3-year longitudinal, real-world, observational, cohort study
Source: PLoS One. 2021 Aug 4;16(8):e0255542. doi: 10.1371/journal.pone.0255542 (PMC8336806; doi:10.1371/journal.pone.0255542)
Supplement: S2 Table — (DOCX) [file pone.0255542.s002.docx]

**S2 Table. The prevalence of co-morbidity in participants before PSM**

|  | **Total cohort**  **(N = 477)** | **Group A (N = 103)**  **(Fractured group)** | **Group B (N = 374)**  **(Not-Fractured group)** |
| --- | --- | --- | --- |
| n (%) | | | |
| **Co-morbidity** | 283 (59.3) | 75 (72.8) | 208 (55.6) |
| **Hypertension** | 117 (24.5) | 29 (28.2) | 88 (23.5) |
| **Gastrointestinal diseases** | 113 (23.7) | 29 (28.2) | 84 (22.5) |
| **Dyslipidemia** | 67 (14.0) | 20 (19.4) | 47 (12.6) |
| **Hepatic diseases** | 38 (8.0) | 13 (12.6) | 25 (6.7) |
| **Diabetes mellitus** | 32 (6.7) | 13 (12.6) | 19 (5.1) |
| **Pulmonary diseases** | 23 (4.8) | 6 (5.8) | 17 (4.5) |
| **Cardiovascular diseases** | 22 (4.6) | 2 (1.9) | 20 (5.3) |
| **Thyroid disorder** | 19 (4.0) | 2 (1.9) | 17 (4.5) |
| **Cerebro-psychiatry**  **diseases** | 16 (3.4) | 5 (4.9) | 11 (2.9) |
| **Hematologic diseases** | 10 (2.1) | 2 (1.9) | 8 (2.1) |
| **Renal disorders** | 8 (1.7) | 3 (2.9) | 5 (1.3) |
