# Supplementary material for: Risk factor analysis of fragility fractures in rheumatoid arthritis: A 3-year longitudinal, real-world, observational, cohort study
Source: PLoS One. 2021 Aug 4;16(8):e0255542. doi: 10.1371/journal.pone.0255542 (PMC8336806; doi:10.1371/journal.pone.0255542)
Supplement: S3 Table — (DOCX) [file pone.0255542.s003.docx]

**S3 Table. The prevalence of co-morbidity in participants after PSM**

|  | **Total participants**  **(n = 309)** | **Group A (n = 103)**  **(Fractured group)** | **Group B (n = 206)**  **(Not-Fractured group)** |
| --- | --- | --- | --- |
| n (%) | | | |
| **Co-morbidity** | 199 (64.4) | 75 (72.8) | 124 (60.2) |
| **Hypertension** | 85 (27.5) | 29 (28.2) | 56 (27.2) |
| **Gastrointestinal diseases** | 81 (26.2) | 29 (28.2) | 52 (25.2) |
| **Dyslipidemia** | 47 (15.2) | 20 (19.4) | 27 (13.1) |
| **Hepatic diseases** | 25 (8.1) | 13 (12.6) | 12 (5.8) |
| **Diabetes mellitus** | 24 (7.8) | 13 (12.6) | 11 (5.3) |
| **Pulmonary diseases** | 17 (5.5) | 2 (1.9) | 15 (7.3) |
| **Cardiovascular diseases** | 16 (5.2) | 6 (5.8) | 10 (4.9) |
| **Thyroid disorder** | 12 (3.9) | 2 (1.9) | 10 (4.9) |
| **Cerebro-psychiatry**  **diseases** | 10 (3.2) | 5 (4.9) | 5 (2.4) |
| **Hematologic diseases** | 6 (1.9) | 2(1.9) | 4 (1.9) |
| **Renal disorders** | 4 (1.3) | 3 (2.9) | 1 (0.5) |
