# Supplementary material for: Risk factor analysis of fragility fractures in rheumatoid arthritis: A 3-year longitudinal, real-world, observational, cohort study
Source: PLoS One. 2021 Aug 4;16(8):e0255542. doi: 10.1371/journal.pone.0255542 (PMC8336806; doi:10.1371/journal.pone.0255542)
Supplement: S4 Table — (DOCX) [file pone.0255542.s004.docx]

S4 Table. Missing data

| Variables | Before PSM | | After PSM | |
| --- | --- | --- | --- | --- |
|  | n | % | n | % |
| Anti-CCP + | 6 | 1.3 | 3 | 1.0 |
| RF + | 20 | 4.2 | 9 | 1.0 |
| HAQ-DI | 20 | 4.2 | 16 | 5.2 |
| DAS-28 (ESR) | 1 | 0.2 | 1 | 0.3 |
| Disease duration (year) | 3 | 0.6 | 3 | 1.0 |
| b/ts DMARDs | 1 | 0.2 | 1 | 0.3 |
| Risk factors in FRAX |  |  |  |  |
| Glucocorticoids | 1 | 0.2 | 0 | 0 |
| Parent fractured hip | 5 | 1.0 | 5 | 1.6 |
| BMD (g/cm^2^) |  |  |  |  |
| Femoral neck | 10 | 2.1 | 8 | 2.6 |
| Hip (total) | 10 | 2.1 | 8 | 2.6 |
| L1~L4 | 7 | 1.5 | 5 | 1.6 |
| Laboratory data |  |  |  |  |
| iPTH (pg/ml) | 6 | 1.3 | 3 | 1.0 |
| 25-OH Vit D (ng/ml) | 68 | 14.3 | 39 | 12.6 |
| Albumin(g/dL) | 9 | 1.9 | 4 | 1.3 |
| BUN (mg/dL) | 6 | 1.3 | 3 | 1.0 |
| Calcium(mg/dL) | 6 | 1.3 | 3 | 1.0 |
| P(mg/dL) | 8 | 1.7 | 3 | 1.0 |
| AST (U/L) | 3 | 0.6 | 0 | 0.0 |
| ALT (U/L) | 7 | 1.5 | 5 | 1.6 |
| ALK-P (U/L) | 11 | 2.3% | 6 | 1.9 |
| Total Bilirubin(mg/dL) | 17 | 3.6 | 8 | 2.6 |
| Platelet(1000/μL) | 1 | 0.2 | 0 | 0.0 |
| FRAX (major) | 10 | 2.1 | 8 | 2.6 |
| FRAX (hip) | 10 | 2.1 | 8 | 2.6 |

Complete data: Age, gender, body weight, body height, BMI, vegetarian, tea, coffee, comorbidity, CRP titer group, ESR, current smoking status, alcohol intake 3 or more units/day, history of previous fracture, secondary osteoporosis, creatinine, white blood count, hemoglobin level

PSM, propensity score matching; anti-CCP, anti-cyclic citrullinated peptide antibody; RF, rheumatoid factor; HAQ-DI, Health Assessment Questionnaire Disease Index; DAS28-ESR, Disease Activity Score of 28 joints using ESR; BMD, bone mineral density; b/tsDMARDs, including anti-TNFa (etanercept, adalimumab, golimumab, certolizumab), anti-IL6 receptor (tocilizumab), CTLA4 analogue (abatacept), anti-CD 20 (rituximab), and JAK inhibitor (tofacitinib); iPTH, intact parathyroid hormone; 25(OH)D, 25-hydroxyvitamin D; BUN, blood urea nitrogen; P, phosphate; AST, aspartate transaminase; ALT, alanine aminotransferase; ALK-P, alkaline phosphatase; FRAX, risk factors of fragility fracture as defined in the FRAX tool
